# Mind the fish: zebrafish as a model in cognitive social neuroscience

**DOI:** 10.3389/fncir.2013.00131

**Published:** 2013-08-08

**Authors:** Rui F. Oliveira

**Affiliations:** ^1^Unidade de Investigação em Eco-Etologia, ISPA – Instituto UniversitárioLisboa, Portugal; ^2^Champalimaud Neuroscience Programme, Instituto Gulbenkian de CiênciaOeiras, Portugal

**Keywords:** social neuroscience, zebrafish, social cognition, cognitive modules, social behavior, social brain

## Abstract

Understanding how the brain implements social behavior on one hand, and how social processes feedback on the brain to promote fine-tuning of behavioral output according to changes in the social environment is a major challenge in contemporary neuroscience. A critical step to take this challenge successfully is finding the appropriate level of analysis when relating social to biological phenomena. Given the enormous complexity of both the neural networks of the brain and social systems, the use of a cognitive level of analysis (in an information processing perspective) is proposed here as an explanatory interface between brain and behavior. A conceptual framework for a cognitive approach to comparative social neuroscience is proposed, consisting of the following steps to be taken across different species with varying social systems: (1) identification of the functional building blocks of social skills; (2) identification of the cognitive mechanisms underlying the previously identified social skills; and (3) mapping these information processing mechanisms onto the brain. Teleost fish are presented here as a group of choice to develop this approach, given the diversity of social systems present in closely related species that allows for planned phylogenetic comparisons, and the availability of neurogenetic tools that allows the visualization and manipulation of selected neural circuits in model species such as the zebrafish. Finally, the state-of-the art of zebrafish social cognition and of the tools available to map social cognitive abilities to neural circuits in zebrafish are reviewed.

## INTRODUCTION: A COGNITIVE APPROACH TO COMPARATIVE SOCIAL NEUROSCIENCE

In social species animals interact frequently with their conspecifics and have to adjust the expression of their social behavior according to previous social experience and to social context. This behavioral flexibility in the social domain (aka social competence; Taborsky and Oliveira, 2012) allows the animal to navigate daily changes in the social environment and should be viewed as an adaptive performance trait that impacts the Darwinian fitness of the animal, for example by allowing it to avoid getting involved in costly social interactions or being ejected from its social group (Oliveira, 2009, 2012). Understanding social competence at the proximate level is a major challenge in contemporary neuroscience. The new field of social neuroscience has emerged in the past two decades in an attempt to understand how biological systems in general, and the brain in particular, implement social behavior on one hand, and how social processes feedback on biological mechanisms and the brain, on the other (Cacioppo and Decety, 2011). One major challenge in this new field is finding the appropriate level of analysis when relating social to biological phenomena. Given the enormous complexity of both the neural networks of the brain and social systems, mapping adaptive social behaviors in the real world onto putative underlying neural circuits in the brain is a daunting task (Adolphs, 2010). One promising approach to this challenge is to use the cognitive level of analysis as an interface level of explanation between brain and behavior, which enables the development of manageable theories of social behavior that can generate testable predictions of observable behavior. Cognition is used here in an information processing perspective, that is as a set of neuronal processes concerned with the acquisition, retention, and use of information, that enables the animal to integrate input information with stored information, when making ecologically relevant decisions (Shettleworth, 2001; Dukas, 2004). These encompass a wide array of cognitive processes such as: perception, learning, memory, attention, and decision making. It should be stressed that the use of the term cognition as proposed here is neither in opposition to association learning explanations of animal behavior in the associative vs. cognitive debate (e.g., Byrne and Bates, 2006; Heyes, 2012a), nor does it equate with intelligence, intentionality or consciousness, as sometimes suggested in anthropomorphic accounts of animal behavior (e.g., Ristau, 1991).

Arguably, it has been proposed that the mechanisms controlling the organism interactions with other behavioral agents (i.e., social interactions) differ from those involved in the interactions of the organism with its physical environment, and therefore the term social cognition has been created to refer specifically to cognitive processes involved in social interactions (e.g., Zuberbuhler and Byrne, 2006). Social phenomena that have been examined under the label of social cognition include recognition of individuals or social categories, social partner preferences, development and management of social relationships (attachment, reconciliation, alliances), triadic relationships (requesting transitive inference), learning new skills from conspecifics (social learning), social coordination, manipulation, and deception, and theory of mind among many others (Jensen et al., 2011). Most research on comparative social cognition has focused mainly on declarative human-like cognitive abilities apparently needed to navigate highly complex social systems such as those of primates (e.g., “theory of mind,” Premack and Woodruff, 1978; Penn and Povinelli, 2007; “Machiavellian intelligence,” Whiten and Byrne, 1988, 1997), and not so much on the basic information processing mechanisms that make up the building blocks of the behavioral control systems involved in social behavior irrespective of its complexity (Barrett et al., 2007). This approach is limiting since highly complex social systems do not necessarily request highly complex individual cognitive abilities, as can be illustrated by insect societies or by elaborated mutualistic relationships in cleaner fish (Chittka and Niven, 2009; Bshary, 2011), and as a consequence most “simple-minded” species have not been considered in comparative studies of social cognition. Moreover, functionally similar social phenomena may rely on different underlying mechanisms in different species (e.g., different cognitive mechanisms underlying transitive inference, see below for details). Therefore, a more productive approach to comparative social cognition would be the adoption of a rationale that can be applied universally across species with varying degrees of complexity of their social structures and that takes into account the underlying mechanisms.

In this paper, I propose a conceptual framework for comparative social neuroscience based on: (1) the identification of the functional building blocks of social behavior and the underlying cognitive mechanisms across different species with varying social systems, and (2) how these information processing mechanisms are inbuilt in the brain, which is viewed as an information processing organ. Following Krogh’s principle, that “for many problems there is an animal on which it can be most conveniently studied” (Krebs, 1975), teleost fish are presented here as a golden model to develop this approach given the diversity of social systems present in closely related species that allows for planned phylogenetic comparisons of cognitive abilities (e.g., MacLean et al., 2012), and the availability of genetic tools that allows the visualization and manipulation of selected neural circuits in model species such as the zebrafish (e.g., Muto and Kawakami, 2011).

## COGNITIVE MODULES OF SOCIAL COMPETENCE

**FIGURE 1:**
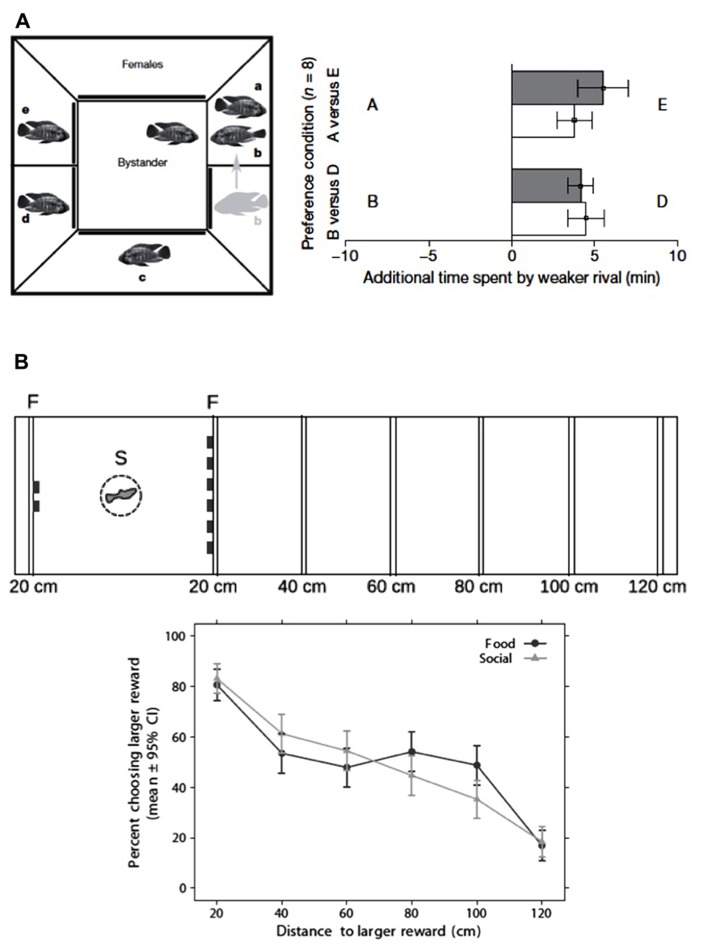
**Examples of two complex social abilities in teleost fish.** **(A)** Transitive inference in a cichlid fish (*Astatotilapia burtoni*; left panel) experimental set-up for transitive inference training; the bystander male is placed in the middle compartment and allowed to observe a sequence of paired fights among neighboring conspecifics; in each fight the scheduled loser male is placed in the territory of the scheduled winner (e.g., A wins over B), so that the following sequence of fights is produced: A > B, B > C, C > D, D > E; in order to test for transitive inference of social dominance focal males were given a preference test between the pairs AE and BD (where A > E and B > D; right panel) focal males prefer to associate with the lower ranking male from each dyad both in familiar (filled bars) and in novel contexts (open bars), suggesting tha males are able to infer the relative rankings (i.e., A > E and B > D) from the observed dominance hierarchy A > B > C > D > E (reproduced with permission from Grosenick et al., 2007). **(B)** Spatial discounting in guppies (*Poecilia reticulata*; upper panel) experimental set-up for the study of spatial discounting; subjects were given a choice between two vs. six items (food items in the food- reward condition and conspecifics in the social-reward condition); the six items (food or conspecifics) were placed at increasing distances (20, 40, 60, 80, 100, 120cm), whereas the two items were always located at 20 cm from the starting place; (lower panel) in choice tests subjects show a preference for the larger reward that decreases with the distance to the larger reward (reproduced with permission from Muhlhoff et al., 2011).

The first step of the conceptual framework proposed here is to identify the information processing problems posed by the social domain of the environment in order to identify the cognitive abilities underlying social skills. For instance, what are the mechanisms required for an individual to tolerate the presence of conspecifics, to recognized different classes of conspecifics and assess their behavior, to use public information available in social environments and to choose the appropriate responses from the available behavioral repertoire? Once we identify these building blocks of social competence we can investigate their phylogenetic distribution and how they map onto neural networks underlying behavior. The putative building blocks of social competence are identified and discussed below (see **Table 1** and **Figure 1** for summary and selected examples in teleost fish, respectively).

**Table 1:** Social skills, their putative underlying cognitive mechanisms and selected examples of their occurrence in teleost fish.

Social skill	Cognitive mechanisms^*^	Species	Reference
**Prosociality**
social reward	Innate response; Selective attention	Zebrafish, *Danio rerio*	Al-Imari and Gerlai (2008)
**Social preferences**
Shoal mate preference	discrimination learning	Zebrafish, *Danio rerio*	Engeszer et al. (2007)
Mate choice	discrimination learning	Peacock blenny, *Salaria pavo*	Fagundes et al. (2007)
Pair bond	recognition learning and social memory	African cichlid, *Tropheus moorii*	Egger et al. (2006)
**Cognitive appraisal**
Predictability	S-S learning	Mozambique tilapia, *Oreochromis mossambicus*	Galhardo et al. (2011)
**Social recognition and memory**
Individual recognition	Single stimulus learning + long-term	Cleaner wrasse, *Labroides dimidiatus*	Tebbich et al. (2002)
Kin recognition	memory	Zebrafish, *Danio rerio*	Gerlach and Lysiak (2006)
Social status		Mozambique tilapia, *Oreochromis*	Barata et al. (2007)
Familiarity		*mossambicus*	
**Social inference**
Social eavesdropping	Selective attention, S–R learning	Siamese fighting fish, *Betta splendens*	Oliveira et al. (1998)
Transitive inference	Associative strength, ordinal representation	African cichlid, *Astatotilapia burtoni*	Grosenick et al. (2007)
Audience effects	Selective attention, S–R learning	Siamese fighting fish, *Betta splendens*	Doutrelant et al. (2001)
Deception	Selective attention, S–R learning	Cleaner wrasse, *Labroides dimidiatus *	Pinto et al. (2011)
**Social learning**
Stimulus enhancement	Single stimulus learning	Zebrafish, *Danio rerio*	Lindeyer and Reader (2010)
Observational conditioning	S–S learning	Zebrafish, *Danio rerio*	Suboski et al. (1990)
Copying	S–R learning	Sailfin molly, *Poecilia latipinna*	Witte and Ryan (2002)
**Intertemporal choice**
Spatial discounting	Reversal learning	Guppies, *Poecilia reticulata*	Muhlhoff et al. (2011)

* Following the terminology used by Shettleworth (2010)

### SOCIAL VALUE AND SOCIAL PREFERENCES

Approach/ avoidance is a basic behavioral mechanism present in all animals. Given that an a priori condition for social groups to form is that individuals show a predisposition to approach conspecifics and tolerate their presence, this prosocial tendency has to overcome the one for social withdrawal. At the cognitive level prosocial behavior relies on a value system that attributes valence (on a negative–positive continuum) and salience (on a low to high continuum) to social agents (Paul et al., 2005), such that conspecifics tend to have reward value (i.e., high salience, positive valence; Thiel et al., 2008) hence eliciting approach responses. However, different conspecifics may pose different challenges/opportunities, hence not all group members are expected to have the same social value. Some might be competitors, others potential partners or mates. These differences in reward value of different conspecifics lead to social preferences (e.g., mate choice preferences; Ryan et al., 2007), which in turn may lead to social bonding, when individuals establish long-lasting relationships.

### COGNITIVE APPRAISAL

Given the wide array of social signals conveyed in multiple sensory modalities it is postulated that a general appraisal mechanism that assesses the valence and salience of social stimuli across different sensory modalities and functional domains must operate. According to this view the evaluation of valence and salience of social information is not just a result of direct effects of perceptual information (e.g., image of conspecific elicits approach), but rather a function of what that perceptual information means to the organism at that moment in time (e.g., image of conspecific is appraised and its valence elicits appropriate response, such as: if dominant avoid; Paul et al., 2005; Mendl et al., 2010). This subjective value of social stimuli is assessed through a set of stimulus evaluation checks which include intrinsic valence of the stimuli, novelty (as defined by suddenness, familiarity, and predictability), prediction error and controllability (Paul et al., 2005; Mendl et al., 2010). Despite the fact that some of these checks have been described in animals (e.g., predictability in fish; Galhardo et al., 2011), a systematic study of stimulus evaluation checks in animals is still lacking. Cognitive appraisal classifies social stimuli in terms of their valence, salience and the organism capacity for control, therefore decoupling stimulus and response and allowing the animal to give a flexible response.

### SOCIAL RECOGNITION AND MEMORY

For the expression of both prosocial behavior and social preferences individuals need to discriminate between classes of social agents, namely conspecifics from heterospecifics and between conspecifics with different social valences. Different forms of social recognition might occur, from individual recognition, where individuals are recognized by unique cues (Tibbetts and Dale, 2007), to the recognition of social classes of individuals, such as kin (Hepper, 1986) or social rank conveyed by status badges (Johnstone and Norris, 1993). Individual recognition is expected to evolve in semi-permanent groups where individuals engage in repeated interactions, since it reduces the costs associated with agonistic interactions and it stabilizes dominance hierarchies (Barnard and Burk, 1979; Pagel and Dawkins, 1997). Any kind of social recognition requires memory for conspecifics so that the acquired discrimination of different individuals or classes of individuals is carried forward in time in a computational way that is accessible for retrieval by the animal at a future time. There is some evidence that social memory is independent of asocial memory. For example, AVP V1b receptor knockout mice have impaired memory for social odors, despite having normal olfactory ability and other memory functions (e.g., spatial memory; Wersinger et al., 2004).

### SOCIAL INFERENCE

In a social group, individuals may gain information by observing social interactions between third parties, thus avoiding the potential costs involved in direct agonistic interactions (McGregor and Peake, 2000). Bystanders may use the information about interacting partners in order to adjust their future behavior in subsequent interactions with the observed individuals (social eavesdropping; Peake, 2005). Similarly, the presence of bystanders may influence current behavior of interacting individuals that will try to manipulate the information available to bystanders (audience effects; Matos and Schlupp, 2005). In social groups information obtained from observing relationships between third parties (e.g., A > B and B > C) can be used to infer unknown relationships among group members (e.g., A > C; Paz-Y-Miño et al., 2004; Grosenick et al., 2007). Therefore, together with individual recognition, transitive inference can stabilize hierarchies in groups with repeated interactions among individuals, since the relative dominance of unfamiliar individuals can be estimated from observing these interacting with familiar ones. Thus, transitive inference (i.e., if A > B and B > C then A > C) is a skill that is expected to develop with increasing social complexity. Indeed, in two independent comparative studies of transitive inference abilities in closely related species differing in sociality, it was found in both cases that the more highly social of the two species performed better in the transitive inference task (corvids: Bond et al., 2003; prosimian primates: MacLean et al., 2008). It should be pointed out that transitive inference can be achieved using different cognitive mechanisms (transfer of associative strength, i.e., value transfer vs. representation of ordinal list), hence it does not necessarily require high-order reasoning abilities (Von Fersen et al., 1991; Allen, 2006); see also (De Lillo et al., 2001) for a description of neural network that solves a transitive inference task using a simple error-correcting rule.

### SOCIAL LEARNING

Public information is readily available in social networks, which allows individuals to acquire adaptive information produced by others without paying the costs typically associated with exploring the environment to learn about its contingencies (e.g., Burns et al., 2011; Mery, 2013). Extracting potentially useful information from observing or interacting with other behavioral agents or their products (aka social learning; Heyes, 1994; Galef and Laland, 2005) has been considered to rely on social-domain specific cognitive modules and in learning mechanisms that are distinct from those used in individual learning by trial and error. However, recent research challenges these assumptions and it has been proposed that asocial and social learning share the same basic learning mechanisms, namely single stimulus learning (e.g., habituation and sensitization vs. local/stimulus enhancement), stimulus–stimulus learning (Pavlovian conditioning vs. observational conditioning), or stimulus–response learning (instrumental learning vs. copying/ imitation; Heyes, 2012b). For example, it has been pointed out that prediction error (i.e., the difference between predicted and obtained outcomes), that is considered to be the learning signal asocial associative learning, is not directly experienced in social learning. However, a new form of observational prediction error has been proposed recently that acts as a learning signal based on externally observed information (e.g., Burke et al., 2010). Social learning is usually use strategically by animals depending on who and when rules, so that they optimize the trade-off between the accuracy and the costs of personal vs. social information (Kendal et al., 2009).

### INTERTEMPORAL CHOICE

In order to maintain social relationships individuals have sometimes to choose a smaller immediate reward to guarantee larger future benefits. This trade-off between two or more pay-offs at different points in time is called temporal discounting. For example, in the cleaning mutualism between the cleaner wrasse (*Labroides dimidiatus*) and its reef fish clients, cleaners feed against their preference (ectoparasites instead clients’ mucus) in order to secure the possibility for numerous future interactions (Grutter and Bshary, 2003), hence exhibiting temporal discounting.

### TRAFFIC RULES

Group-living animals need to synchronize and coordinate their behavior in order to maintain the functionality of the social group (e.g., in collective motion a group member needs to move with the group in order to keep the benefits of group membership such as predator avoidance). This behavioral synchronization and coordination of individuals within social groups has lead to the emergence of collective patterns that can be impressive, such as the aerial movements of bird flocks, or the spatial behavior of insect swarms, fish schools, ungulate herds, and even human crowds. In most of these cases complex collective patterns can be explained by individual decision-making rules and by the way information flows between group members (Sumpter, 2006; Couzin, 2009). For example, fish schools can be modeled using individual-based models that follow as few as three rules: avoid individuals that are too close, align with individuals at intermediate distance, and move toward those further away (Parrish and Turchin, 1997; Parrish and Viscido, 2005). Therefore, simple heuristics at the level of the individual may explain the emergence of self-organized social patterns without the need of complex cognitive abilities. This does not mean, however, that the cognitive abilities mentioned above are not needed for optimized social behavior in varying social environments.

More complex social systems are predicted to impose a higher cognitive demand, hence recruiting quantitatively more resources of the abovementioned social skills or promoting qualitative progress of new social skills (e.g., “theory of mind” in humans and questionably in non-human primates; Byrne and Bates, 2010). Evidence supporting this view came from comparative work in primates that established an association between brain size (in particular neocortex), social complexity (as measured by social group size or by occurrence of long-term relationships), and social skills (as a proxy of cognitive complexity), which has been interpreted as evidence for positive selection on executive brain size driven by social complexity (aka “Social brain hypothesis;” Dunbar, 1998; Reader and Laland, 2002; Byrne and Corp, 2004; Dunbar and Shultz, 2007). Although originally developed in primates (Dunbar, 1992; Barton, 1996), the social brain hypothesis has been extended to other taxa, including non-primate mammals (e.g., Perez-Barberia et al., 2007), birds (e.g., Burish et al., 2004; Emery et al., 2007), fish (e.g., Pollen et al., 2007; Gonzalez-Voyer et al., 2009) and insects, on which the relationship between sociality and a brain area size was first described (see review in Lihoreau et al., 2012). However, it has been pointed out that apparently complex social skills may require little information-processing capabilities (Chittka and Niven, 2009) and that even qualitative enhancements in information-processing behind behavioral innovations may be achieved with minor changes in the connectivity of neural networks (see below). Therefore, future research on the co-evolution of brain and social cognition/behavior should move beyond comparative analyses of brain size and focus on unraveling the neural circuitry underlying specific social cognitive abilities.

## FUNCTIONAL ARCHITECTURE OF THE SOCIAL BRAIN

The second step of the proposed conceptual framework is to map the cognitive processes involved in social competence onto the brain. There is ample evidence indicating that complex cognitive functions are associated with distributed brain networks, rather than with single brain regions, such that their behavioral manifestations are better reflected by the overall pattern of activation across the different loci of the network than by the activity of any of the single nodes (McIntosh, 2000; Sporns, 2010). These networks are also dynamic so that each node (i.e., brain region) may participate in multiple cognitive functions by rapid functional connectivity reconfigurations (Sporns, 2010). The combination of functional specialization in domain-specific modules with the integration at the neural network level provides coherence to mental states and to behavioral (motor) decision making, allowing for the expression of complex and flexible behavior. Each functional network may exhibit a variety of states, as defined by the configuration of activated nodes, each of which expressing the network encoded knowledge regarding a specific input. The existence of social domain-specific modules within these networks has been demonstrated both at the sensory and central levels as can be illustrated by the parallel stream of social odor processing by the mammalian vomeronasal system relative to asocial odors processed by the main olfactory system (Døving and Trotier, 1998), or by the specialized face recognition areas in the brains of humans, macaques (*Macaca mulatta*) and sheep (Kendrick and Baldwin, 1987; Kanwisher and Yovel, 2006; Tsao et al., 2006, 2008).

**FIGURE 2:**
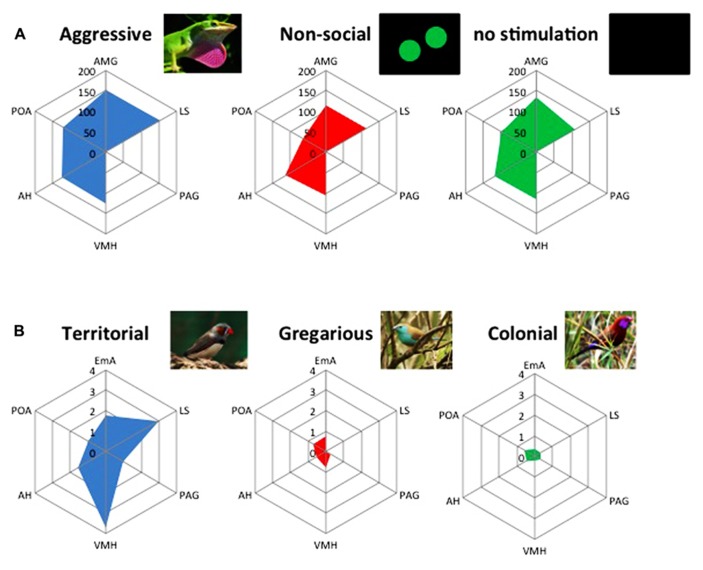
**The brain social behavior network (BSBN) presents different activation states associated with the expression of different social behaviors both at the intra- (A)** and interspecific level **(B)**. **(A)** Pattern of activation of the BSBN in the green anole lizard (*Anolis carolinensis*), as measured by the activity of cytochrome oxidase (μmol/min/g tissue), elicited by the exposure to videoplaybacks of an aggressive display, a non-social stimulus (moving balls) or no stimulation (adapted from Yang and Wilczynski, 2007). **(B)** Divergent patterns of activation of the BSBN, as measured by the expression of the immediate early gene c-fos (arbitrary units), elicited by the exposure to a conspecific in closely related songbird species with divergent social systems: territorial (violet-eared waxbill, *Uraeginthus granatina*), gregarious (Angolan blue waxbill, *Uraeginthus angolensis*), highly colonial species (zebra finch, *Taeniopygia guttata*). Note the higher activation of EmA, AH, VMH, and LS in the territorial species (adapted from Goodson et al., 2005). EmA, extended medial amygdala; LS, lateral septum; PAG, periaqueductal gray; VMH, ventromedial hypothalamus; AH, anterior hypothalamus; POA, pre-optic area.

Recently, the occurrence of an evolutionary conserved social decision-making network in vertebrates has been proposed, based on conserved patterns of expression of developmental genes and neurochemical systems in the telencephalon (O’Connell and Hofmann, 2011, 2012). This vertebrate social decision-making network would be composed of two interconnected sister networks: the basal forebrain reward system and the social behavior network (*sensu*; Goodson, 2005). The reward system would provide value information to other domain-specific modules in the network so that the valence and salience of social stimuli can be integrated in social decision-making, allowing for the reinforcement of adaptive behaviors through natural rewards (Kelley and Berridge, 2002). The social behavior network would be involved in the regulation of multiple forms of social behavior and includes the extended medial amygdala, the lateral septum, the preoptic area, the anterior hypothalamus, the ventromedial hypothalamus, and the periaqueductal gray in mammals and their homologs in non-mammals (Newman, 1999; Goodson, 2005; Goodson and Kabelik, 2009; O’Connell and Hofmann, 2011; see **Figure 2**). Functional evidence for the presence of the social behavior network is difficult to obtain since it requires the simultaneous recording of neural activity in multiple brain regions. Given the difficulty of obtaining large-scale electrophysiological recordings indirect measures of neural activity, such as the expression of immediate early genes (e.g., *c-fos, egr-1*) or the activity of cytochrome oxidase, in relation to the expression of different social behaviors, have been used to test this hypothesis. In recent years this type of studies has been accumulating evidence in favor of the social brain network hypothesis. In the African cichlid *Astatotilapia burtoni* subordinate males given the opportunity to rise in social rank show higher expression of immediate early genes in all nodes of the social behavior network when compared either to stable subordinate or dominant males (Maruska et al., 2013). In the green anole lizard (*Anolis carolinensis*) repeated exposure to video-playbacks of aggressive displays of conspecific males induced changes in functional connectivity within the network (Yang and Wilczynski, 2007). And in estrildid finches, different nodes of the network are differentially activated in response to the presence of a conspecific, in a way that is related to inter-specific differences in sociality (Goodson et al., 2005). Despite this evidence for the association of this brain network with social behavior, a systematic approach to the study of the relationship between specific social phenomena (e.g., individual recognition, social inference, etc.) and network state, that would potentially allow the identification of specific social cognitive modules and their integration, is still missing. So far the study of these processes in relation to large-scale brain activity has been mainly restricted to humans and other primates for which functional brain imaging techniques (e.g., MRI, PET) are available (e.g., Sallet et al., 2011; Kumaran et al., 2012). However, given the unique role of the neocortex in human and non-human primate social cognition the relevance of this type of data for testing the wider social brain network hypothesis outlined above has been limited (Adolphs, 2009).

At the evolutionary level the combination of functional segregation and integration in neural networks also provides a simple explanation for qualitative enhancements in information-processing leading to behavioral innovations with gains in flexibility, that may coexist with ancestral stereotyped responses. For example, in honeybees vertical functional modules, such as those specialized in processing conspecific odors, provide rapid and stereotyped responses, while central integration across multiple interconnected domain-specific modules provides novel and adaptive solutions (Menzel and Giurfa, 2001).

Finally, it must be stressed that evidence supporting the occurrence of this putative social behavior network does not mean that that the nodes of this network are exclusively involved in social decision-making. On the contrary, it is expected each node to be shared by multiple brain networks (e.g., stress and social behavior networks).

## TELEOSTS AS MODELS FOR COMPARATIVE SOCIAL COGNITION

A successful comparative research program in cognitive social neuroscience has two key requirements: (1) the possibility for cladistic research on the evolution of social behavior and cognition aiming to uncover how pre-existing cognitive modules may evolve quantitatively (e.g., increase in memory storage capacity) and how networks may be reconfigured leading to the emergence of qualitatively new solutions to adaptive problems; and (2) the possibility for reductionist research on the mapping of cognitive function into neural networks, which requires model organisms with appropriate social behavior and with an available “tool box” for the analysis of neural circuits. Therefore, the combination of comparative behavioral work on selected species in naturalistic settings covering the expected diversity in cognitive abilities with neuroethological research on a phylogenetically related model organism is a promising approach. Teleost fish fulfill both requirements and therefore they offer an excellent opportunity to fulfill such a research program on comparative cognitive social neuroscience.

First, they offer a unique possibility for planned phylogenetic comparisons on social skills and underlying cognitive modules. With over 29,000 species described so far, teleost fish are the most diverse of the vertebrate taxa, and this diversity also translates into a wide variation within closely related groups of species in modes of social organization (e.g., variation in mating systems and parental care type in African cichlids, Machado et al., 2009). Fish also excel in social plasticity, as can be illustrated by the profound behavioral and phenotypic changes induced by the social environment, which ranges from fish of different social status displaying different neurobehavioral profiles to socially driven sex-change (Godwin, 2010; Fernald, 2012). Complex social behavior is also present among teleost fish, as is the case of transitive inference shown by cichlid fish (Grosenick et al., 2007), or strategic behavior, including deception, punishment, reconciliation, partner choice, manipulation, and social prestige, displayed by the obligatory cleaning wrasse (*Labroides dimidiatus*) on the context of cleaning mutualism (Bshary, 2011). So complex forms of social behavior are present in fish and they offer ample opportunity for comparative work both at the inter- and intra-specific levels.

Second, a number of model organisms have been developed among teleost fish (e.g., zebrafish, medaka) for which neurobiological and genetic tools are becoming increasingly available. Among the current teleost model organisms zebrafish offers the best conditions for research in social neuroscience due to a combination of relevant social behavior with availability of relevant tools for studying brain function in relation to behavior.

## SOCIAL ZEBRAFISH

Zebrafish are highly social animals that live in groups with structured social relationships including shoaling, dominance hierarchies, and territoriality (Spence et al., 2008; Spence, 2011). Furthermore, social behavior in zebrafish shows considerable flexibility as recently shown by the occurrence of acute winner and loser effects (i.e., winner/loser effects; Oliveira et al., 2011), where short-term social interactions are effective in inducing changes in social behavior that are paralleled by massive changes in the profile of gene expression in the brain (Oliveira et al., unpublished data). This richness and flexibility of social behavior predicts that at least some of the social cognitive modules discussed above must be present in zebrafish. Below the available evidence for the occurrence of some of these modules in zebrafish will be discussed.

### SOCIAL VALUE IN ZEBRAFISH

The sight of conspecifics has rewarding value in zebrafish and can be used as a reinforcer in an associative learning task (Al-Imari and Gerlai, 2008). Given the rewarding value of conspecifics it is not surprising that zebrafish form aggregations (shoals) from early in development and that shoaling behavior, as measured by the cohesion of the social aggregation (i.e., distance between each pair of shoal members), increases with age (Engeszer et al., 2007; Buske and Gerlai, 2011). Shoal cohesion varies with social context, increasing in the presence of a predator and decreasing during feeding, which is coherent with the function of shoals in reducing predation risk and enhancing foraging efficiency (Miller and Gerlai, 2007). The maturation of shoaling during development is paralleled by an increase in whole brain dopaminergic and serotonergic activity (Buske and Gerlai, 2012). However, the exposure to conspecific images only induces an increase in brain dopamine, but not in serotonin levels, suggesting a specific involvement of the dopaminergic system in social reward in zebrafish (Saif et al., 2013). This result is in agreement with the well established role of dopamine in the reward circuitry of mammals (Schultz, 2010). However, in anamniotes a midbrain dopaminergic population similar to the ventral tegmental area (VTA), that plays a key role in the mammalian mesolimbic reward system, is missing (Smeets et al., 2000), and the identification of a homologous dopamine reward circuit in fish has remained elusive for many years. The identification of dopaminergic cell groups in the ventral diencephalon projecting to the subpallium (Rink and Wullimann, 2001, 2002), raised the hypothesis that this ascending dopaminergic pathway could be homologous to the mammalian mesostriatal pathway. However, recent data on the expression of developmental factors in larval zebrafish made clear that the ventral diencephalic dopaminergic neurons, that have ascending projections to the telencephalon (i.e., dopaminergic groups DC2 and DC4), are homologous to the A11 mammalian diencephalic dopaminergic cell group (Lohr et al., 2009), rather than to the midbrain mammalian dopaminergic group (i.e., A10). Morever, a detailed projectome of the dopaminergic circuitry in zebrafish showed that most subpallial dopaminergic inputs originate in a local subpallial system that also connects to the ventral telencephalon (Tay et al., 2011). In summary, a the ventral diencephalic-subpallial dopamine system has been characterize in zebrafish that is a good candidate for the dopamine reward system in fish. However, it cannot be seen as homologous to the mammalian dopaminergic mesolimbic pathway, which apparently emerged later in the evolution of dopaminergic modulatory systems (Yamamoto and Vernier, 2011).

### SOCIAL PREFERENCES IN ZEBRAFISH

Although conspecifics act as social rewards zebrafish are not equally attracted to all conspecifics, exhibiting shoaling preferences that emerge during the juvenile phase (Engeszer et al., 2007). These shoaling preferences are visually mediated, so that when given a choice between shoal mates with different coloration patterns, individuals prefer to shoal with those sharing the same coloration pattern as the fish with whom they were raised (Engeszer et al., 2007). Once established shoaling preference remains stable and it is not reversed by changing their social environment (Engeszer et al., 2007). In accordance to this finding, once adults wild type zebrafish do not exhibit a shoaling preference based on visual cues for different phenotypic variants (e.g., leopard danios; Spence and Smith, 2007); transgenic red fluorescent Glofish (Snekser et al., 2006). Other characteristics of the shoal than the phenotype of shoal mates are also relevant for the expression of shoaling preferences in zebrafish, that tend to prefer larger and more active shoals (Pritchard et al., 2001; Ruhl and McRobert, 2005). In mammals social preferences and social bonding are known to be moderated by oxytocin and arginine-vasopressin (Donaldson and Young, 2008). Interestingly, it has been shown that their homologs in fish, isotocin and arginine-vasotocin, respectively, also regulate social preference in zebrafish (Braida et al., 2012), suggesting a conserved mechanism for prosocial behavior involving these two neuropeptides. Social preferences can also be expressed in the sexual context, as mate choice preferences according to which individuals do not mate randomly but prefer males with specific characteristics (Spence and Smith, 2006). Female mating preferences for larger males have been described in zebrafish (Pyron, 2003). Given that fin size can increase perceived body size the preference of female zebrafish for longer fins as also been tested but yielded negative results (Kitevski and Pyron, 2003; Gumm et al., 2009).

### SOCIAL RECOGNITION IN ZEBRAFISH

As mentioned before a prerequisite for individuals to express social preferences is the ability to recognize individuals or classes of individuals. Zebrafish use both visual and olfactory cues in social recognition. Studies on zebrafish reared in social isolation and on cross-reared intra-specific phenotypes showed that visually mediated species recognition is based on a mechanism of phenotype matching against a learned template early in life (McCann and Matthews, 1974; McCann and Carlson, 1982; Engeszer et al., 2004). Olfaction also plays a role in species recognition as well as in kin recognition in zebrafish, again through a process of phenotype matching (Gerlach and Lysiak, 2006). Olfactory kin recognition is based on imprinting with a 24 h critical period on day 6 post-fertilization during which exposure to kin necessary and sufficient (Gerlach et al., 2008). Neither exposure to own chemical cues nor exposure to non-kin in the critical period results in imprinting. Although individual recognition has not been investigated yet in zebrafish, the occurrence of dominance hierarchies in both sexes (Grant and Kramer, 1992; Delaney et al., 2002; Spence and Smith, 2005; Paull et al., 2010), suggests that it may be present.

### COGNITIVE APPRAISAL IN ZEBRAFISH

Cognitive appraisal and cognitive bias are recent research areas that only now are starting to be explored in zebrafish. In our lab we have collected preliminary evidence suggesting that in dyadic agonistic interactions it is the perception that the individual has of the event rather than its objective structure that triggers the physiological and genomic responses differentially observed in winners and losers (Oliveira et al., unpublished data). These results are in accordance with previous work on cichlid fish that showed that ambiguous agonistic interactions between fish and their own image on a mirror (i.e., where the expression of aggressive behavior is decoupled from the experience of gaining or losing social status) failed to elicit the physiological responses observed in winners and losers of real opponent fights (Oliveira et al., 2005).

### SOCIAL LEARNING IN ZEBRAFISH

So far the use of public information in zebrafish has been documented mainly in the context of response to aversive stimuli. A first suggestion of social learning in zebrafish comes from data showing that groups of zebrafish learn an avoidance response to an electric shock faster than single individuals (Gleason et al., 1977). However, the better performance while in a social group may be explained by other mechanism, including motivational factors related to the stress of being in isolation. In another set of studies social facilitation of fear response to a predator cue was established (Suboski et al., 1990; Hall and Suboski, 1995). Like many other ostariophysian fishes zebrafish release an alarm substance to the water when injured which causes a fright response in other fish (Waldman, 1982; Speedie and Gerlai, 2008). This innate fear response can be conditioned by pairing a conditioned stimulus (CS; e.g., innocuous odor or red light) with the exposure to the alarm substance unconditioned stimulus (US; Suboski et al., 1990; Hall and Suboski, 1995). Conditioned individuals can be subsequently used as demonstrators to naïve fish in trained-naïve mixed groups in the presence of the CS alone (i.e., red light in the absence of the alarm substance). In these conditions all fish of the mixed groups exhibited the alarm response indicating social transmission of the conditioned response from the demonstrators to the naïve fish. Moreover, when sorted out from the mixed groups, naïve individuals kept the response to the CS, indicating the acquisition of the response to the predator cue by observational conditioning in naïve individuals (Suboski et al., 1990; Hall and Suboski, 1995). More recently, it has been shown that zebrafish can learn escape routes from trained demonstrators, and that the presence of demonstrators in groups of naïve individuals increased the escape response (i.e., escaped faster) from an approaching trawl. Moreover, observers successfully became demonstrators for further groups of naïve fish and escape responses were experimentally propagated across three generations of social learning (Lindeyer and Reader, 2010). Interestingly, route traditions (i.e., preference for a particular escape route) were not kept along the chain of social transmission, suggesting a mechanism of social facilitation that increases escape response without learning the specific route followed by the demonstrator. Social learning in zebrafish may also be impacted by attributes of the demonstrator. Zebrafish shoals are structured social networks with different individuals having a differential involvement in social interactions (Vital and Martins, 2011), so that central individuals in the network (aka Keystone individuals; Sih et al., 2009) can be recognized. Such keystone individuals play important roles in social groups, acting as learning models and as leaders in group movement (King and Cowlishaw, 2009; Bode et al., 2011). In a recent study keystone and non-key (i.e., less central in the social network) individuals in zebrafish shoals were identified, individually trained in an aversive response task and returned to their shoal. Shoals with trained keystone individuals escaped aversive stimuli more rapidly than those with trained non-key individuals, supporting the hypothesis that social roles play a critical role in social learning also in zebrafish. Apart from the social learning abilities described above, zebrafish also exhibit a wide range of asocial learning abilities in different ecological domains (e.g., Gerlai, 2011; Karnik and Gerlai, 2012), therefore offering the possibility for contrasting the mechanisms underlying learning in the social and physical domains.

### INTERTEMPORAL CHOICE IN ZEBRAFISH

Temporal discounting has not been studied in fish so far. However, spatial discounting (i.e., when animals choose between smaller and closer vs. larger and distant rewards) for social rewards has been recently demonstrated in guppies (Muhlhoff et al., 2011). Both types of discounting require impulse control which is usually tested using reversal learning paradigms. A recent study established the occurrence of reversal learning in zebrafish, hence opening the possibility for the occurrence of intertemporal choices in this species (Parker et al., 2012).

### TRAFFIC RULES IN ZEBRAFISH

Fish groups can be classified either as “shoals” or as “schools”, depending on the degree of synchronization and polarization among group members. Thus, shoals are aggregations of individuals [with four body lengths (BLs) commonly used as a criterion for shoal membership in cyprinid species; Pitcher et al., 1983], whereas schools are highly synchronized and coordinated shoals with polarized orientation of individuals (Pitcher and Parrish, 1993). Both types of groups are present in zebrafish, with schools being faster and less dense than zebrafish shoals, and occurring at lower densities (Miller and Gerlai, 2012). Zebrafish groups may vary in size and are characterized by a high degree of changes in individual relative position within the group and by motion pathways with a high rate of changes in direction (Miller and Gerlai, 2007, 2008, 2011; see also Viscido et al., 2004 for data on the giant danio, *Danio aequipinnatus*). The average distance among zebrafish shoal mates is approximately of 20 cm, and it responds to environmental factors, increasing in the presence of food and predators to over 30 cm (Miller and Gerlai, 2007). Considering that adult zebrafish BL varies between 3 and 4 cm, the average distance between any shoal mates corresponds to 5–6.6 BLs, which is above the proposed threshold of four BLs for shoal membership. However, this distance to the nearest neighbor is stable over time suggesting temporal shoal cohesion in zebrafish (Miller and Gerlai, 2007, 2008, 2011). Although many traffic rules have been developed to explain schooling and shoaling behavior in fish (e.g., Parrish and Turchin, 1997; Parrish and Viscido, 2005), only recently one of these models have been tested in zebrafish. This model showed that zebrafish follows a simple rule in social decision-making based on Bayesian estimation that uses the behaviors of other individuals to improve the estimation (Arganda et al., 2012), therefore confirming the idea that simple heuristics may explain apparently complex collective behavior also in zebrafish.

## TOOLS FOR STUDYING BRAIN FUNCTION IN ADULT ZEBRAFISH

A significant number of genetic and neuroanatomy tools and resources are becoming available for zebrafish, making it a tractable species to study brain behavior relationships. Detailed brain atlases are now available for adult zebrafish (Wullimann et al., 1996), and homologies, based on topological and functional data, between zebrafish and mammalian brain areas have been established (Wullimann and Mueller, 2004). More recently, magnetic resonance imaging (MRI) techniques were developed for zebrafish and a detailed MRI three-dimensional atlas is now available for adult zebrafish (Ullmann et al., 2010c,d). The use of MRI will potentially allow non-invasive acquisition of brain morphological data and provides more precise estimates of brain area size than those obtained by classical histological methods, which are prone to tissue deformations due to dissection or histological processing (Ullmann et al., 2010b). This technique has a high potential not only for intra-specific studies with a model organism like the zebrafish, but also for inter-specific comparative studies of brain volumes in relation to social behavior (Pollen et al., 2007). As an example of the rapid development of the field recently 3D MRI atlases became available for two more fish species (i.e., Tilapia, *Oreochromis mossambicus*: Simões, 2012; and barramundi, *Lates calcarifer*: Ullmann et al., 2010a).

The functional study of neural circuits in zebrafish has benefited from the development of optogenetic and transgenic techniques that together allow the close monitoring of activity in neural networks and experimental gain and loss of function manipulations to assess causal relationships between specific neural patterns and specific behaviors (Baier and Scott, 2009; Portugues et al., 2013). Imaging of neural activity in the brain of both larvae and adults (explants in the later case) has been achieved using genetically encoded calcium indicators, of which successive versions of GCaMP have been the more widely used (Baier and Scott, 2009; Portugues et al., 2013). The use of these fluorescent reporters requires the restrain of the animal during image acquisition which limits the behavioral tasks that can be investigated. In order to overcome this limitation a virtual reality system has been recently developed for zebrafish larvae, in which the larvae is stationary but the putative motor output is recorded from the motor neuron axons in the tail and is used in real time to drive movement in the virtual environment (Ahrens et al., 2012). Gain and loss of function studies at the level of cell type or small groups of neurons have used opsin photoswitchable probes, such as channelrhodopsin (ChR2) and halorhodopsin (NpHR), that activate neurons in a reversible way in response to light pulses of specific wave-lengths (e.g., Douglass et al., 2008; Arrenberg et al., 2009). Loss of function studies have also used chemical or photo inducible probes (e.g., Tetanus toxin, nitroreductase, Killer red) to selectively silence specific neurons in neural circuits (e.g., Koide et al., 2009). Viral transfection and transgenesis have been used as two alternative ways to restrict the expression of the abovementioned reporters and manipulators of neural activity to specific components of the neural networks (Zhu et al., 2009). In particular the Gal-UAS binary transgenic system has been used successfully to specify genetically targeted cell populations and to relate them to specific behaviors, even in adults (e.g., Agetsuma et al., 2010; Okamoto et al., 2012; Muto et al., 2013).

In contrast to the optogenetics toolbox available to study brain function in larval zebrafish, the available tools for adults are far more limited. With the ossification of the skull during development the efficacy of optogenetic techniques decreases and at most they can still be used *in vivo* in the juvenile phase. On the other hand, the repertoire of social behavior is very limited in larvae, whose ethogram is limited to locomotor action patterns involved in swimming and in prey capture (Budick and O’Malley, 2000). Thus, a major challenge for future research on zebrafish social neuroscience is to try to match in development the efficient use of optogenetic tools with the availability of relevant behavior. For doing this the period during which optogenetic tools can be efficiently used will have to be moved forward in development on one hand, and on the other a detailed characterization of the ontogeny of the cognitive abilities underlying social skills is needed in order to identify how early different cognitive abilities can be successfully studied in zebrafish. Whenever the use of the zebrafish optogenetic toolbox becomes available to the study of social abilities, it will offer an unprecedented opportunity to characterize the neural networks underlying social cognitive modules, and to experimentally manipulate particular nodes of the network and infer their potential role on domain-specific modules on a vertebrate model. Until then mapping of the neural activity that underlies cognitive processes in adult zebrafish is still recurring to the expression of immediate early genes as markers of neural activity (e.g., Lau et al., 2011).

## PROSPECTS

The field of social neuroscience has emerged in the past two decades as a vibrant and very successful branch of twenty-first century neuroscience, and understanding the relationship between social cognition and the social brain became a hot topic. However, different research traditions coexist within the field with parallel research programs. Researchers from a more Biomedical or Human Psychology background have been mainly focused on human social behavior and on translational research (e.g., Cole et al., 2007; Norman et al., 2012). Primatologists and comparative psychologists have focused on testing the occurrence of “uniquely human” cognitive abilities (e.g., theory of mind, deception, cooperation) in a small subset of “cognitively complex” animals such as primates, dolphins, and more recently corvids and elephants (Connor, 2007; Byrne and Bates, 2009, 2010; Bugnyar, 2011). Finally, behavioral ecologists and neuroethologists focus their research on understanding the functional value and the underlying neural mechanisms of social cognitive skills in a wide range of “simple-minded” animals (e.g., insects, fish), that are seen as adaptive traits that help survival and successful breeding (Bshary et al., 2002; Chittka and Niven, 2009; Bshary, 2011; Chittka and Skorupski, 2011; Taborsky and Oliveira, 2012). In recent years significant efforts to integrate these different research streams have been made, namely through the organization of thematic discussions and meetings. Although challenging due to the multitude of concepts, methodologies, and conflicting points of view (e.g., associative vs. cognitive explanations of animal behavior), an integration of these traditions would be a major breakthrough for the understanding of the basic proximate and ultimate mechanisms of social cognition and behavior. The present paper was written in that spirit and would finish by identifying some outstanding questions and future challenges in the field that would benefit from an integrated approach and that the adoption of the conceptual framework proposed here will hopefully help to address:

(1) The creation of a common cognitive lexicon and taxonomy so that clear concepts are shared – the occurrence of complex social behaviors does not necessarily request complex cognitive abilities; therefore functionally similar cognitive abilities that rely on different underlying mechanisms should be recognized (e.g., transitive inference and transitive inference-like abilities).
(2) The modularity of social cognition – experimental approaches are needed to clarify the conflicting results between intra- and inter-specific analyses of social cognitive modularity. Group living species whose social behavior can be easily recreated in captivity, with relatively short inter-generation times (when compared to the 6 weeks of the mice that is the standard model for behavioral genetics) and easy to breed in the lab, can be successfully used in the future to address this question using experimental evolution paradigms, as suggested by a recent study on artificial selection for larger brains in guppies (*Poecilia reticulata*) that had an impact on cognitive skills within two generations (Kotrschal et al., 2013).
(3) The distributed nature of information processing in neural networks should be taken into account when trying to map cognitive processes onto brain activity; therefore comparative analyses should move beyond the comparison of brain sizes and focus on inter-specific convergence/divergence in functional connectivity in the social decision making neural network.
  In answering all these questions I foresee a relevant role for species that offer the possibility to integrate imaging of brain activity with relevant behavioral tasks, as is the case of zebrafish.

## Conflict of Interest Statement

The author declares that the research was conducted in the absence of any commercial or financial relationships that could be construed as a potential conflict of interest.
